# Crystal structure of diethyl 2,2′-[((1*E*,1′*E*)-{[(1*R*,4*R*)-cyclo­hexane-1,4-di­yl]bis­(aza­nylyl­idene)}bis­(methanylyl­idene))bis­(1*H*-pyrrole-2,1-di­yl)]di­acetate

**DOI:** 10.1107/S2056989015002674

**Published:** 2015-02-13

**Authors:** Jasim Alshawi, Muoayed Yousif, Karzan H. Zangana, Inigo J. Vitorica Yrezabal, Richard Winpenny, Mohamad J. Al-Jeboori

**Affiliations:** a Department of Chemistry, College of Education for Pure Science, University of Basrah, Iraq; b School of Chemistry, University of Manchester, Oxford Road, Manchester M13 9PL, England; c Department of Chemistry, College of Education (Ibn Al-Haitham) for Pure Science, University of Baghdad, Iraq

**Keywords:** crystal structure, Schiff base, bis­pyrrole, C—H⋯O hydrogen bonding

## Abstract

The whole mol­ecule of the title compound, C_24_H_32_N_4_O_4_, is generated by inversion symmetry. The cyclo­hexane ring adopts a chair conformation and the conformation about the C=N bonds is *E*. The pyrrole rings have an *anti* confirmation with respect to the cyclo­hexane moiety and the ethyl acetate groups have extended conformations. In the crystal, mol­ecules are linked by pairs of C—H⋯O hydrogen bonds forming chains, enclosing *R*_2_^2^(10) ring motifs with inversion symmetry, propagating parallel to the (101) plane.

## Related literature   

For general background on the applications of Schiff bases and the use of pyrrole compounds, see: Köse *et al.* (2015 ▸); Trofimov *et al.* (2015 ▸). For the synthesis of di­pyrrole Schiff bases ligands, see: Meghdadi *et al.* (2010 ▸); Munro *et al.* (2004 ▸). For the synthesis of pyrrole ester precursors, see: Koriatopoulou *et al.* (2008 ▸); Singh & Pal (2010 ▸). For the preparation of Schiff bases, see: Yang *et al.* (2004 ▸); Ourari *et al.* (2013 ▸).
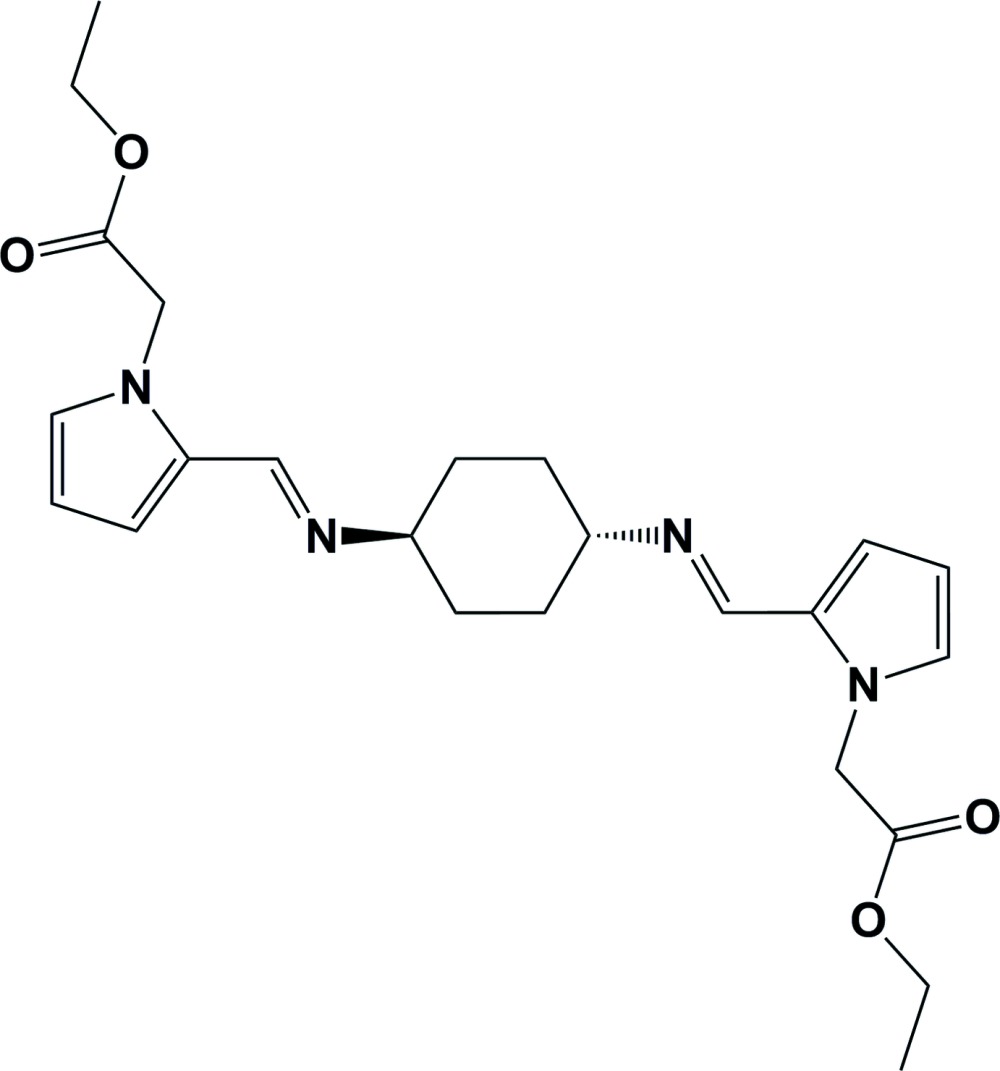


## Experimental   

### Crystal data  

C_24_H_32_N_4_O_4_
*M* *_r_* = 440.54
Triclinic, 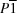

*a* = 8.5531 (6) Å
*b* = 8.8379 (7) Å
*c* = 9.6492 (9) Å
α = 115.166 (9)°
β = 92.105 (7)°
γ = 113.288 (8)°
*V* = 587.68 (10) Å^3^
*Z* = 1
Mo *K*α radiation
μ = 0.09 mm^−1^
*T* = 150 K
0.4 × 0.3 × 0.3 mm

### Data collection  

Agilent SuperNova (single source at offset, Atlas) diffractometer
Absorption correction: multi-scan (*CrysAlis PRO*; Agilent, 2013 ▸) *T*_min_ = 0.933, *T*_max_ = 1.000
4657 measured reflections
2734 independent reflections
1827 reflections with *I* > 2σ(*I*)
*R*_int_ = 0.028

### Refinement  

*R*[*F* ^2^ > 2σ(*F* ^2^)] = 0.055
*wR*(*F* ^2^) = 0.124
*S* = 1.07
2734 reflections
146 parameters
H-atom parameters constrained
Δρ_max_ = 0.18 e Å^−3^
Δρ_min_ = −0.25 e Å^−3^

### 

Data collection: *CrysAlis PRO* (Agilent, 2013 ▸); cell refinement: *CrysAlis PRO*; data reduction: *CrysAlis PRO*; program(s) used to solve structure: *SHELXS2014* (Sheldrick, 2008 ▸); program(s) used to refine structure: *SHELXL2014* (Sheldrick, 2015 ▸); molecular graphics: *PLATON* (Spek, 2009 ▸); software used to prepare material for publication: *SHELXL2014* and *PLATON*.

## Supplementary Material

Crystal structure: contains datablock(s) Gobal, I. DOI: 10.1107/S2056989015002674/su5080sup1.cif

Structure factors: contains datablock(s) I. DOI: 10.1107/S2056989015002674/su5080Isup2.hkl

**Fig. 1.**
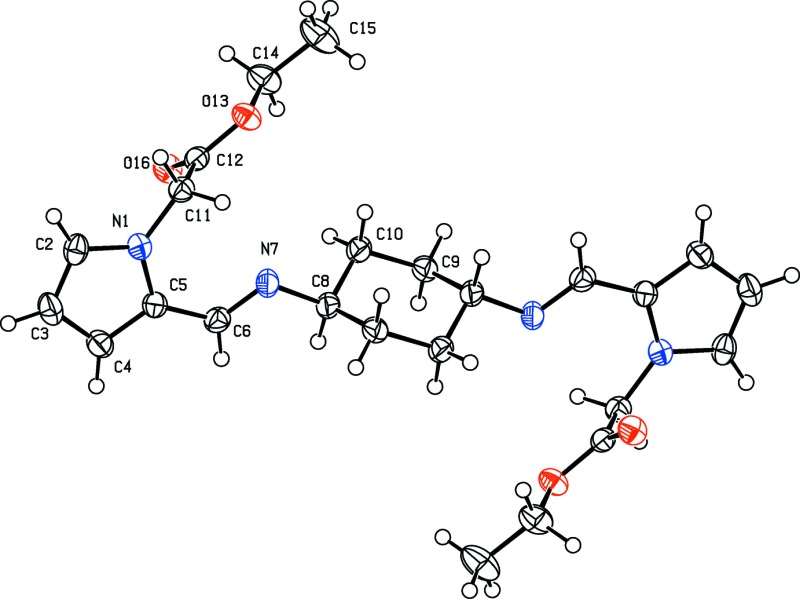
A view of the molecular structure of the title compound. Displacement ellipsoids are drawn at the 50% probability level.

**Fig. 2.**
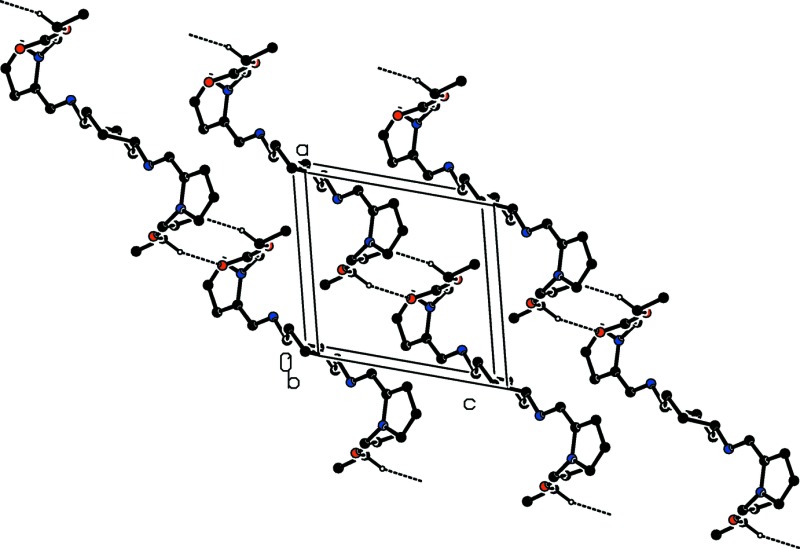
A view along the b axis of the crystal packing of the title compound. The C—H···O hydrogen bonds are drawn as dashed lines (see Table 1 for details; H atom not involved in hydrogen bonding have been omitted for clarity).

. DOI: 10.1107/S2056989015002674/su5080fig1.tif
A view of the mol­ecular structure of the title compound. Displacement ellipsoids are drawn at the 50% probability level.

b . DOI: 10.1107/S2056989015002674/su5080fig2.tif
A view along the *b* axis of the crystal packing of the title compound. The C—H⋯O hydrogen bonds are drawn as dashed lines (see Table 1 for details; H atom not involved in hydrogen bonding have been omitted for clarity).

CCDC reference: 1048163

Additional supporting information:  crystallographic information; 3D view; checkCIF report

## Figures and Tables

**Table 1:** Hydrogen-bond geometry (, )

*D*H*A*	*D*H	H*A*	*D* *A*	*D*H*A*
C14H14*A*O16^i^	0.97	2.50	3.317(3)	142

Symmetry code: (i) 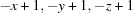.

## supplementary crystallographic information

### S1. Synthesis

The title compound was prepared in a two step procedure:

Synthesis of ethyl (2-formyl-1*H*-pyrrole-1-yl)-acetate (L): prepared by reported procedures (Koriatopoulou *et al.*, 2008; Singh & Pal, 2010) as follows: To a mixture of 1*H*-pyrrole-2-carbaldehyde (1.00 g, 10.51 mmol), K_2_CO_3_ (2.90g, 21.02 mmol) and (2.64 g, 10.51 mmol) of 18-crown-6 in dry 1,4-dioxane (20ml), was added drop wise a solution of ethyl bromo­acetate (2.00 g, 12 mmol) in dry 1,4-dioxane (20 ml), over a period of 30 min. The reaction mixture was allowed to reflux under a nitro­gen atmosphere for 6 h, and then the solvent was removed under reduced pressure. Water (50ml) was added to the residue, and the mixture was extracted with ethyl acetate (3 × 15ml). The combined organic layers were washed with brine (15 ml), and then dried over Na_2_SO_4_. The solvent was removed under reduced pressure, and the oily residue was purified by flash chromatography with an eluent mixture (33% ethyl acetate / hexane), giving compound (L) as a yellow oil product (yield: 0.75 g, 75%). NMR data (p.p.m), δH: (500 MHz, CDCl_3_): 1.20 (3H, t, C12—H), 4.15 (2H, q, C11—H), 4.97 (2H, s, C8—H), 6.21 (1H, t, C3—H), 6.84 (1H, d, C4—H), 6.90 (1H, d, C2—H) and 9.45 (1H, s, C6—H); δC (125.75 MHz, CDCl3), 14.13 C12, 50.25 C8, 61.63 C11, 110.20 C3, 124.61 C4, 131.71 C5 and 132.10 C2. C=O for the carboxyl­ate moiety at 168.37 (C12) and at 179.74 for C6. The positive ES mass spectrum at m/z = 182.4 (M+H)^+^ (62%) for C_9_H_11_NO_3_, requires = 181.1. The other peaks detected at m/z =153.4 (100%), 109.3 (6%), 95 (9%) and 67 (4%) correspond to [M—CH_2_CH_3_]^+^, [M-(CH_2_CH_3_+CO_2_)]^+^, [M-(CH_2_CH_3_+CO_2_+CH_2_)]^+^ and [M-(CH_2_CH_3_+CO_2_+CH_2_+CO)]+, respectively. IR (ATR cm^-1^): 1650 ν(C=O) aldehyde moiety. 1710 ν(C=O) ester group.

Synthesis of the title Schiff-base: performed using conventional procedures (Yang *et al.*, 2004; Ourari *et al.*, 2013). To a mixture of L (1.81 g, 10 mmol) in ethanol (20 ml) with 3 drops of glacial acetic acid, a solution of 1,4-di­amino­cyclo­hexan (0.57 g, 5 mmol) in ethanol (20ml) was added drop wise over a period of 20 min. The reaction mixture was allowed to reflux for 3h, and then cooled to room temperature. A white precipitate was collected by filtration and recrystallised from ethanol (yield: 1.09g, 60%). Crystals were obtained by slow evaporation of a solution in methanol/acetone. NMR data (p.p.m), δH (500 MHz, CDCl_3_): 1.19 (6H, t,C15, 15–H), 1.47 (C10, 10- ,4H, q), δH = 1.67 (C9, 9-, 4H, q), 2.94 (2H, p, C8, 8–H), 4.10 (4H, q,C14, 14–H), 5.03 (C11, 11–H, 4H, s), 6.11 (2H, t, C3, 3–H), 6.38 (2H, d, C4, 4–H), 6.61 (2H, d, C2, 2–H) and 8.07 (2H, s, C6, 6–H); δC (125.75 MHz, CDCl3), 14.28 (C15, 15-). 32.61 (C10, 10- and C19, 9- ), 51.13 (C11, 11-), 61.07 (C14, 14- ), 68.8 (C8, 8-), 108.53 (C2, 2-), 116.58 (C5, 5-), 127.69 (C3, 3-), 129.93 (C4, 4-), 150.04 (C6, 6-), C=O 169.37 (C12, 12-). The positive ES mass spectrum at m/z = 441.52 (M+H)^+^ (100%) for C_24_H_32_N_4_O_4_, requires = 440.24. The other peaks detected at m/z = 412.42 (5%), 383 (3%), 295.19 (9%) and 267.1 (4%) correspond to [M—CH_2_CH_3_]^+^, [M-(2CH_2_CH_3_)]^+^, [M-(2CH_2_CH_3_+2CO_2_)]^+^ and [M(2CH_2_CH_3_+2CO_2_+2CH_2_)]^+^, respectively. IR (ATR, cm^-1^): 1580 (C=N), 1630 (C=O).

### S2. Refinement

Crystal data, data collection and structure refinement details are summarized in Table 2. All H atoms were placed in calculated positions and treated as riding atoms: C—H = 0.95 - 0.99 Å with U_iso_(H) = 1.5U_eq_(C) for methyl H atoms and = 1.2U_eq_(C) for other H atoms.

### S3. Comment

The whole molecule of the title compound, Fig.1, is generated by inversion symmetry. The cyclohexane ring adopts a chair conformation and the conformation about the C═N bonds is *E*. The pyrrole rings crystallize in the anti-confirmation with respect to the cyclohexane moiety and the ethyl acetate moieties have extended conformations.

In the crystal, molecules are linked by pairs of C—H···O hydrogen bonds forming chains, enclosing *R*^2^_2_(10) ring motifs with inversion symmetry, propagating parallel to plane (101); see Table 1 and Fig. 2.

Infrared spectrum indicated typical absorbance bands of the functional –C═ N and carbonyl –C═O at 1580 and 1630 cm^-1^, respectively. The positive ES mass spectrum of the bis Schiff-base showed a parent ion peak at m/*z* = 441.52 (*M*+H)^+^, corresponding to C_26_H_32_N_4_O_4_, for which the required value is 440.24. The N7═C6 bond distance [1.270 (2) Å] is shorter than the N2—C8 bond distance [1.458 (2) Å], indicating a double bond order. However, the N1—C5 bond distance [1.384 (2) Å] indicates resonance has occurred in the pyrrole system between the lone pair electron of the nitrogen atom and the pyrrole ring.

### Crystal data

**Table:**

C_24_H_32_N_4_O_4_	*Z* = 1
*M**_r_* = 440.54	*F*(000) = 236
Triclinic, *P*1	*D*_x_ = 1.245 Mg m^−^^3^
Hall symbol: -P 1	Mo *K*α radiation, λ = 0.71073 Å
*a* = 8.5531 (6) Å	Cell parameters from 1515 reflections
*b* = 8.8379 (7) Å	θ = 3.3–26.7°
*c* = 9.6492 (9) Å	µ = 0.09 mm^−^^1^
α = 115.166 (9)°	*T* = 150 K
β = 92.105 (7)°	Block, colourless
γ = 113.288 (8)°	0.4 × 0.3 × 0.3 mm
*V* = 587.68 (10) Å^3^	

### Data collection

**Table:**

Agilent SuperNova (single source at offset, Atlas) diffractometer	2734 independent reflections
Radiation source: SuperNova (Mo) X-ray Source	1827 reflections with *I* > 2σ(*I*)
Mirror monochromator	*R*_int_ = 0.028
Detector resolution: 10.37 pixels mm^-1^	θ_max_ = 29.4°, θ_min_ = 2.9°
ω scans	*h* = −11→11
Absorption correction: multi-scan (*CrysAlis PRO*; Agilent, 2013)	*k* = −10→12
*T*_min_ = 0.933, *T*_max_ = 1.000	*l* = −13→8
4657 measured reflections	

### Refinement

**Table:**

Refinement on *F*^2^	Primary atom site location: structure-invariant direct methods
Least-squares matrix: full	Hydrogen site location: inferred from neighbouring sites
*R*[*F*^2^ > 2σ(*F*^2^)] = 0.055	H-atom parameters constrained
*wR*(*F*^2^) = 0.124	*w* = 1/[σ^2^(*F*_o_^2^) + (0.0363*P*)^2^ + 0.1039*P*] where *P* = (*F*_o_^2^ + 2*F*_c_^2^)/3
*S* = 1.07	(Δ/σ)_max_ < 0.001
2734 reflections	Δρ_max_ = 0.18 e Å^−^^3^
146 parameters	Δρ_min_ = −0.25 e Å^−^^3^
0 restraints	

### Special details

**Table:**

Geometry. All e.s.d.'s (except the e.s.d. in the dihedral angle between two l.s. planes) are estimated using the full covariance matrix. The cell e.s.d.'s are taken into account individually in the estimation of e.s.d.'s in distances, angles and torsion angles; correlations between e.s.d.'s in cell parameters are only used when they are defined by crystal symmetry. An approximate (isotropic) treatment of cell e.s.d.'s is used for estimating e.s.d.'s involving l.s. planes.

### Fractional atomic coordinates and isotropic or equivalent isotropic displacement parameters (Å^2^)

**Table:**

	*x*	*y*	*z*	*U*_iso_*/*U*_eq_	
O13	0.46898 (16)	0.46998 (17)	0.19574 (15)	0.0317 (3)	
O16	0.63475 (17)	0.71083 (17)	0.43494 (15)	0.0338 (4)	
N1	0.67517 (19)	0.9650 (2)	0.32094 (18)	0.0274 (4)	
N7	0.87176 (19)	0.7539 (2)	0.17968 (18)	0.0313 (4)	
C12	0.5599 (2)	0.6517 (3)	0.3013 (2)	0.0270 (4)	
C6	0.9477 (2)	0.9265 (3)	0.2821 (2)	0.0297 (5)	
H6	1.0695	0.9854	0.3163	0.036*	
C5	0.8559 (2)	1.0361 (2)	0.3483 (2)	0.0275 (4)	
C10	0.9066 (2)	0.4733 (2)	0.1181 (2)	0.0311 (5)	
H10A	0.7851	0.3992	0.0575	0.037*	
H10B	0.9087	0.4935	0.2252	0.037*	
C11	0.5474 (2)	0.7689 (2)	0.2290 (2)	0.0279 (4)	
H11A	0.5653	0.7192	0.1233	0.033*	
H11B	0.4302	0.7597	0.2201	0.033*	
C2	0.6355 (3)	1.1083 (3)	0.4039 (2)	0.0317 (5)	
H2	0.5229	1.0970	0.4061	0.038*	
C9	1.0097 (2)	0.3654 (3)	0.0461 (2)	0.0307 (5)	
H9A	0.9545	0.2438	0.0420	0.037*	
H9B	1.1280	0.4334	0.1127	0.037*	
C8	0.9819 (2)	0.6620 (2)	0.1201 (2)	0.0304 (5)	
H8	1.1007	0.7412	0.1897	0.036*	
C4	0.9269 (3)	1.2260 (3)	0.4484 (2)	0.0340 (5)	
H4	1.0459	1.3098	0.4864	0.041*	
C3	0.7880 (3)	1.2712 (3)	0.4833 (2)	0.0377 (5)	
H3	0.7980	1.3898	0.5483	0.045*	
C14	0.4577 (3)	0.3383 (3)	0.2513 (2)	0.0400 (5)	
H14A	0.4025	0.3576	0.3392	0.048*	
H14B	0.5743	0.3562	0.2869	0.048*	
C15	0.3512 (3)	0.1451 (3)	0.1175 (3)	0.0544 (7)	
H15A	0.4111	0.1242	0.0342	0.082*	
H15B	0.2388	0.1314	0.0787	0.082*	
H15C	0.3349	0.0551	0.1534	0.082*	

### Atomic displacement parameters (Å^2^)

**Table:**

	*U*^11^	*U*^22^	*U*^33^	*U*^12^	*U*^13^	*U*^23^
O13	0.0352 (8)	0.0259 (7)	0.0293 (8)	0.0091 (6)	0.0017 (6)	0.0141 (6)
O16	0.0378 (8)	0.0349 (7)	0.0245 (8)	0.0133 (6)	0.0036 (6)	0.0140 (6)
N1	0.0295 (8)	0.0254 (8)	0.0269 (9)	0.0133 (7)	0.0060 (6)	0.0114 (7)
N7	0.0275 (8)	0.0302 (8)	0.0281 (9)	0.0150 (7)	0.0050 (7)	0.0054 (7)
C12	0.0238 (10)	0.0303 (10)	0.0253 (10)	0.0116 (8)	0.0079 (8)	0.0124 (8)
C6	0.0255 (10)	0.0337 (10)	0.0262 (10)	0.0119 (9)	0.0061 (8)	0.0126 (9)
C5	0.0291 (10)	0.0282 (10)	0.0231 (10)	0.0120 (8)	0.0062 (8)	0.0115 (8)
C10	0.0250 (10)	0.0340 (10)	0.0249 (10)	0.0115 (9)	0.0054 (8)	0.0083 (8)
C11	0.0250 (10)	0.0305 (10)	0.0265 (10)	0.0120 (8)	0.0052 (8)	0.0129 (8)
C2	0.0412 (11)	0.0355 (10)	0.0299 (11)	0.0252 (10)	0.0127 (9)	0.0178 (9)
C9	0.0259 (10)	0.0280 (10)	0.0300 (11)	0.0104 (8)	0.0039 (8)	0.0089 (8)
C8	0.0204 (9)	0.0302 (10)	0.0293 (11)	0.0116 (8)	0.0034 (7)	0.0050 (8)
C4	0.0361 (11)	0.0269 (10)	0.0308 (11)	0.0105 (9)	0.0062 (9)	0.0106 (9)
C3	0.0519 (13)	0.0269 (10)	0.0341 (12)	0.0207 (10)	0.0109 (10)	0.0118 (9)
C14	0.0480 (13)	0.0336 (11)	0.0396 (13)	0.0130 (10)	0.0065 (10)	0.0240 (10)
C15	0.0690 (16)	0.0316 (11)	0.0498 (15)	0.0101 (12)	−0.0021 (12)	0.0217 (11)

### Geometric parameters (Å, º)

**Table:**

O13—C12	1.336 (2)	C6—C5	1.441 (2)
O13—C14	1.448 (2)	C5—C4	1.375 (2)
O16—C12	1.203 (2)	C10—C9	1.522 (2)
N1—C5	1.384 (2)	C10—C8	1.522 (3)
N1—C11	1.452 (2)	C2—C3	1.365 (3)
N1—C2	1.363 (2)	C9—C8^i^	1.526 (3)
N7—C6	1.270 (2)	C8—C9^i^	1.526 (3)
N7—C8	1.458 (2)	C4—C3	1.405 (3)
C12—C11	1.506 (3)	C14—C15	1.490 (3)
			
C12—O13—C14	116.15 (15)	C4—C5—C6	127.83 (17)
C5—N1—C11	126.32 (15)	C8—C10—C9	111.74 (14)
C2—N1—C5	108.70 (15)	N1—C11—C12	112.58 (15)
C2—N1—C11	124.84 (15)	N1—C2—C3	108.91 (17)
C6—N7—C8	117.71 (15)	C10—C9—C8^i^	111.47 (17)
O13—C12—C11	109.47 (16)	N7—C8—C10	109.64 (14)
O16—C12—O13	124.75 (19)	N7—C8—C9^i^	109.49 (17)
O16—C12—C11	125.74 (17)	C10—C8—C9^i^	110.20 (15)
N7—C6—C5	123.74 (17)	C5—C4—C3	107.99 (18)
N1—C5—C6	124.91 (15)	C2—C3—C4	107.14 (17)
C4—C5—N1	107.27 (16)	O13—C14—C15	107.88 (18)
			
O13—C12—C11—N1	−166.07 (14)	C11—N1—C5—C6	3.5 (3)
O16—C12—C11—N1	16.3 (3)	C11—N1—C5—C4	−176.36 (17)
N1—C5—C4—C3	0.4 (2)	C11—N1—C2—C3	176.43 (17)
N1—C2—C3—C4	−0.3 (2)	C2—N1—C5—C6	179.33 (19)
N7—C6—C5—N1	6.4 (3)	C2—N1—C5—C4	−0.6 (2)
N7—C6—C5—C4	−173.7 (2)	C2—N1—C11—C12	−110.7 (2)
C12—O13—C14—C15	179.68 (16)	C9—C10—C8—N7	176.06 (15)
C6—N7—C8—C10	134.27 (18)	C9—C10—C8—C9^i^	55.5 (2)
C6—N7—C8—C9^i^	−104.71 (19)	C8—N7—C6—C5	178.46 (18)
C6—C5—C4—C3	−179.5 (2)	C8—C10—C9—C8^i^	−56.2 (2)
C5—N1—C11—C12	64.5 (2)	C14—O13—C12—O16	2.0 (3)
C5—N1—C2—C3	0.6 (2)	C14—O13—C12—C11	−175.70 (15)
C5—C4—C3—C2	0.0 (2)		

Symmetry code: (i) −*x*+2, −*y*+1, −*z*.

### Hydrogen-bond geometry (Å, º)

**Table:**

*D*—H···*A*	*D*—H	H···*A*	*D*···*A*	*D*—H···*A*
C14—H14*A*···O16^ii^	0.97	2.50	3.317 (3)	142

Symmetry code: (ii) −*x*+1, −*y*+1, −*z*+1.
