# Supplementary figures and images for: Crystal structure of diethyl 2,2′-[((1E,1′E)-{[(1R,4R)-cyclo­hexane-1,4-di­yl]bis­(aza­nylyl­idene)}bis­(methanylyl­idene))bis­(1H-pyrrole-2,1-di­yl)]di­acetate
Source: Acta Crystallogr E Crystallogr Commun. 2015 Feb 13;71(Pt 3):o165–6. doi: 10.1107/S2056989015002674 (PMC4350751; doi:10.1107/S2056989015002674)

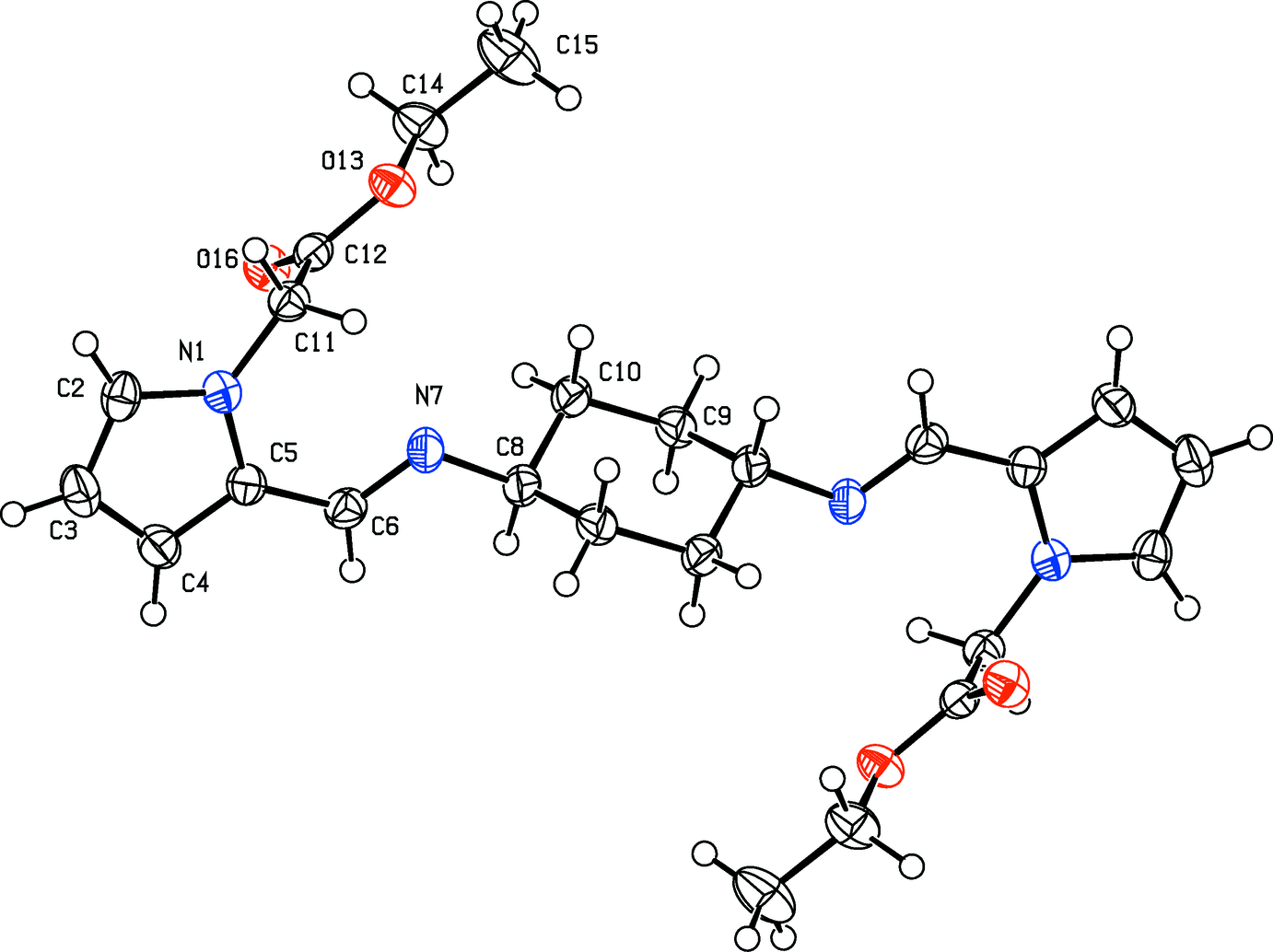

Supplement: Supplementary file 3 [file e-71-0o165-fig1.tif]

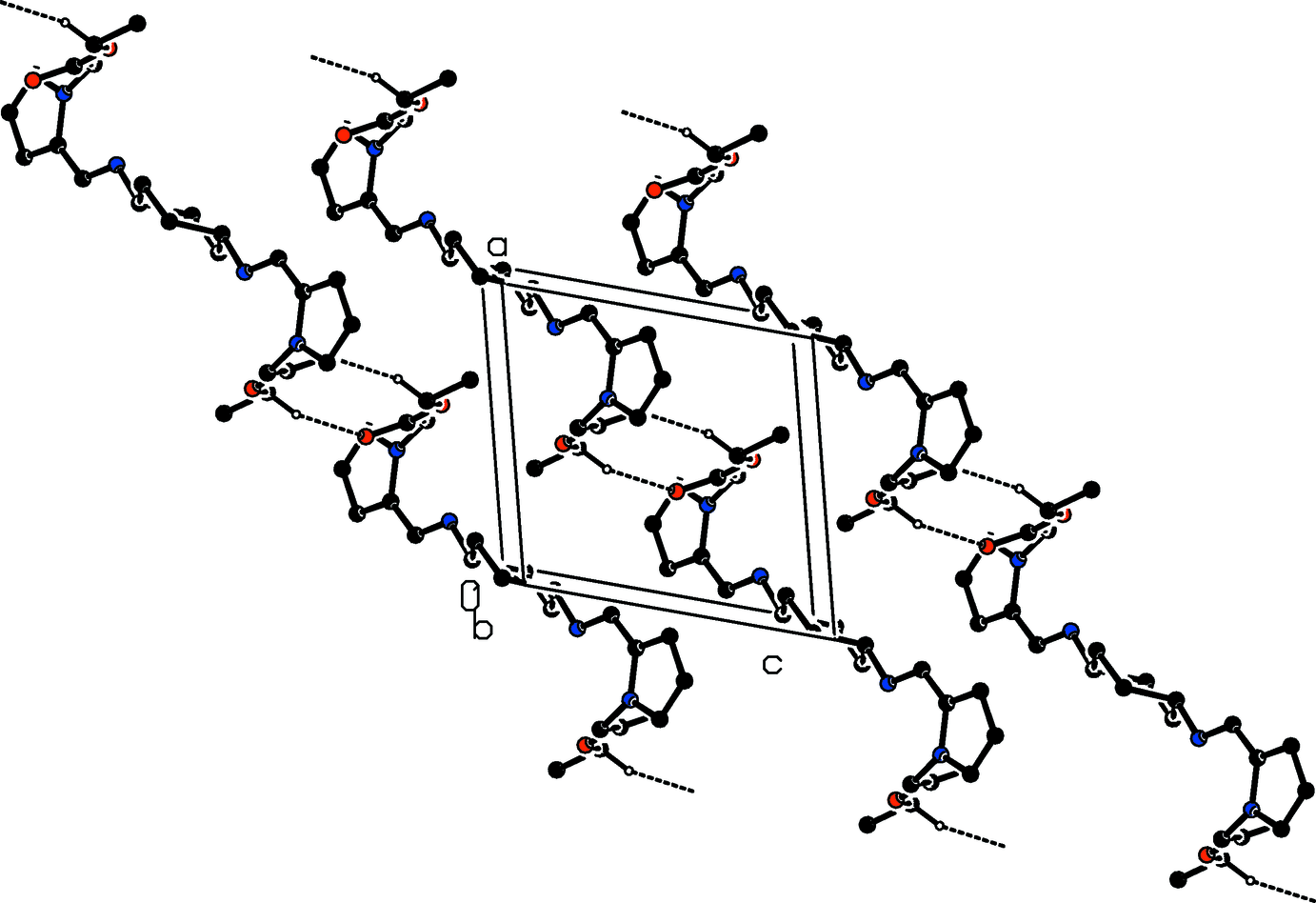

Supplement: Supplementary file 4 [file e-71-0o165-fig2.tif]
